# Echoes of the Month

**Published:** 1924-06

**Authors:** 


### ECHOES OF THE MONTH.

### NEW LIGHT DEPARTMENT AT ALTON.

The installation of a new light department at the Lord Mayor Treloar's Home for Cripples at Alton, shortly to be opened by the Duke of York, is a valuable addition to the wonderful work which is being carried on among suffering children under the control of Sir Henry Gauvain. The essential feature of the installation is the possibility of using rays of different wave-lengths, either separately or in combination, for treatment in various tuberculous conditions. In no institution have such facilities for varying sources of radiation been made available as at Alton. The effects of these various rays are too numerous to mention. On lupus, that most distressing and disfiguring of diseases, the results are especially brilliant and obvious. Septic wounds, constantly discharging, which have failed to respond to any other form of treatment, frequently dry up and heal, leaving supple and almost invisible scars. Sequestra, or fragments of dead diseased bones, are often spontaneously and painlessly extruded without surgical intervention. Hair may be made to grow on patches which previously were bald, and many other effects of this marvellous use of healing light could be described. Perhaps the most striking of all the effects of this treatment is that on the general condition of afflicted patients, whose wasted and emaciated bodies become developed and robust.

### OUR CHANGING FACES.

A curious change is taking place in the growth processes affecting the face, declared Professor Sir Arthur Keith, lecturing on "Changes Affecting the Jaws and Teeth of English People," at a meeting of the Dental Board of the United Kingdom at the Royal College of Surgeons. The bone of the mouth, he added, was growing downwards and carrying with it the teeth, as a result of which there had been an increase of over half-an-inch in the length of the modern face. These changes were also affecting the upper bones of the face, giving it a downward tendency. They correlated with the change in our society, brought about by the industrial revolution, and he believed that they were due to the absence of light in our big cities. It was noticeable that only a small proportion of the population was being affected, namely, about 20 to 30 per cent., and that only a certain proportion of the teeth were affected. The contracted palate which was so noticeable in the modern skull was brought about by these changes.

### ANTS DEGENERATE THROUGH ALCOHOL.

Lecturing on Ants before the Royal Institution, Mr. Balfour Browne explained that among the guests that the ant harbours in its cummunities are creatures that excrete alcohol, and he further explained that the habit of using this alcohol is as deleterious to the ant communities as it is generally stated to be in human civilisation. Degeneration occurs among the ants, and by use or abuse of these particular guests the young emerge as degenerate and undeveloped females. Mr. Browne described the elaborate way in which the ants look after the aphides, which have often been described as the ants' cows—they secrete a "honey-dew"—how they take them into their colonies during the cold weather and carefully plant them out on cultivated crops, so that they can have them at hand for use in warmer weather. It is the civilisation of the ant that makes it so difficult in many instances for the farmer to compete against the aphides, which he naturally regards as a mere pest.

### WORTHY OF THE PROPHET HOSEA.

Dr. R. A. Gibbons, gynæcologist to the Grosvenor Hospital, in a paper on the "State Certificates of Marriage," read before the Eugenics Education Society, put forward the suggestion that the State should prevent the marriage of those whose health, mental condition, or inability to earn a livelihood made the production of a healthy family an impossibility. He said that the State might withhold a certificate in cases of persons suffering from venereal diseases, tubercle, mental deficiency, or genuine poverty. Young men married when quite unable to keep a wife, with the result that children, when born, were underfed, neglected, and finally thrown on to the public funds. The State, he suggested, might insist on each individual taking out a policy of insurance, and if the medical examination proved unsatisfactory the certificate could be withheld and marriage postponed. Sir John Bland-Sutton described Dr. Gibbons's paper as an indictment of modern matrimony worthy of the prophet Hosea. Eugenics, it appeared, was going to arrange an entirely new kind of marriage.

### ROOF "BATHS."

"Every house built in the future will have a sunbath on the roof, for people will come to look on sunbaths as an elementary necessity of life, the same as a bath," declared Mr. Clement Jeffery, in a lecture on "The Sun Cure," at 74, Grosvenor Street recently. He added: "It is quite feasible. Even to-day suburban houses ought to be built with flat roofs, so that tenants can have a sunbath before their day's work."

### TRIUMPH OF "THE WILL TO LIVE."

Professor Lazarus Barlow, in his speech at the opening meeting of the British Empire Cancer campaign, mentioned two interesting cases of recovery. In one case, a woman went out completely well and with no trace of cancer after three years in hospital; in another and still more remarkable case a woman recovered after suffering from cancer for eighteen years, fourteen of which she had spent in hospital. In neither case was there any "cure." Medicine has no cure. They were examples of what Professor Barlow called the power of the body "to hold an equilibrium" against the disease. In other words, it was an example of the "will to live" triumphing over disease.

### A BELGIAN HOSPITAL FOR MONGOLIA.

M. Joseph Tchang, a young Chinese doctor, embarked recently at Marseilles to return to China, says the *Libre Belgique*. M. Tchang has been staying in Belgium for two years to study. He has been following Dr. Schockaert's lectures at Louvain and has also studied at the Institut Pasteur de Bruxelles. With him are going the two Belgian monks of Saint Augustin, belonging, as he does, to the Belgian Hospital founded in Mongolia by the missionaries of Scheut. Two Belgian doctors, Drs. Nachtergael and Kaisin, are already working in this hospital. They are in the middle of an immense tract of country wherein there is no other doctor to be found. Besides caring for the sick they spend much time in bacteriological research and in training both men and women to be nurses.

### A BANTING FOUNDATION.

In recognition of the international services to medicine and to humanity of Dr. F. G. Banting, Mr. H. C. Best and those who co-operated with them in the discovery and development of insulin, the Banting Research Foundation has been established under the direction of men of distinction in Canada. A campaign will be conducted for public subscriptions in Canada and the United States with the object of raising at least £100,000 and with an objective of £200,000. The purposes of the Foundation are to provide, in the first instance, further funds for the support of the Banting-Best Chair of Medical Research at the University of Toronto; to establish a fund for the adequate financial support of such scientific workers as may have proposed definite problems of medical research, and for whom funds are not otherwise available.

### MORE EDUCATION IN HEALTH MATTERS.

Speaking at Newcastle on "Women in the Local Government Service," Miss Cooper-Hodgson, Superintendent Health Visitor for the County of Durham, urged that there should be more women in the Local Government service. She said it seemed to her that nobody objected to a woman working. The objection was to her getting a decently-paid job. Equal pay for equal work was a very thorny problem. The Rathbone scheme of pooling wages was worth considering in connection with equal pay for equal work. Emphasising the need for education on health matters, Miss Cooper-Hodgson said the enormous amount of ill-health that resulted from eating the wrong kind of food was not realised. She also referred to the wrong way in which many working people spent their money, and declared that it could not be denied that if they had all the money spent on drink and betting in Northumberland and Durham they could have settled the housing problem over and over again. "We still have not got sense enough to see what a 'mug's' game betting is," added the speaker. "It is a perfectly appalling business."

